# Association Between Prior Calcium Channel Blocker Use and Mortality in Septic Patients: A Meta-Analysis of Cohort Studies

**DOI:** 10.3389/fphar.2021.628825

**Published:** 2021-05-25

**Authors:** Xianfei Ding, Yuqing Cui, Huoyan Liang, Dong Wang, Lifeng Li, Quancheng Kan, Lexin Wang, Tongwen Sun

**Affiliations:** ^1^ General Intensive Care Unit, The First Affiliated Hospital of Zhengzhou University, Henan Key Laboratory of Critical Care Medicine, Zhengzhou Key Laboratory of Sepsis, Henan Engineering Research Centre for Critical Care Medicine, Zhengzhou, China; ^2^ Internet Medical and System Applications of National Engineering Laboratory, Cancer Center, The First Affiliated Hospital of Zhengzhou University, Zhengzhou, China; ^3^ Department of Pharmacy, The First Affiliated Hospital of Zhengzhou University, Zhengzhou, China; ^4^ School of Biomedical Sciences, Charles Sturt University, Wagga Wagga, NSW, Australia

**Keywords:** calcium channel blocker, sepsis, septic shock, mortality, systematic review, meta-analysis, prior

## Abstract

**Background:** The aim of this study was to comprehensively review the literature and synthesize the evidence concerning the relationship between prior calcium channel blocker (CCB) use and mortality in patients with sepsis.

**Methods:** The Medical Literature Analysis and Retrieval System Online (MEDLINE), Excerpta Medica database (EMBASE), Cochrane CENTRAL, and Web of Science databases were searched from their inception to April 9, 2020. Cohort studies related to prior calcium channel blocker use in patients with sepsis were analyzed. Pairs of reviewers independently screened the studies, extracted the data, and assessed the risk of bias. The primary outcome of 90-days mortality or secondary outcome of short-term mortality, including 30-days, Intensive Care Unit (ICU), and in-hospital mortality, were analyzed. Heterogeneity among studies was assessed using the *I* ^2^ statistic and was considered moderate if *I* ^2^ was 50–75% and high if *I* ^2^ was ≥75%. Random-effects models were used to calculate the pooled odds ratios (ORs) and 95% confidence intervals (CIs). The quality of the studies was evaluated with the Newcastle-Ottawa Scale (NOS). Sensitivity analyses were performed to examine the robustness of the results.

**Results:** In total, 639 potentially relevant studies were identified, and the full texts of 25 articles were reviewed. Ultimately, five cohort studies involving 280,982 patients were confirmed to have a low risk of bias and were included. Prior CCB use was associated with a significantly lower 90-days mortality in sepsis patients [OR, 0.90 (0.85–0.95); *I* ^2^ = 31.9%]. Moreover, prior CCB use was associated with a significantly reduced short-term mortality rate in septic shock patients [OR, 0.61 (0.38–0.97); *I* ^2^ = 62.4%] but not in sepsis patients [OR, 0.83 (0.66–1.04); *I* ^2^ = 95.4%].

**Conclusion:** This meta-analysis suggests that prior CCB use is significantly associated with improved 90-days mortality in sepsis patients and short-term mortality in septic shock patients. This study provides preliminary evidence of an association between prior CCB use and mortality in sepsis patients.

## Background

Sepsis is defined as a life-threatening disorder of organ function caused by a dysregulated host response to infection (Singer et al., 2016). Global epidemiological data suggest that sepsis is a major public health issue and remains a primary cause of mortality and critical illness; sepsis affects millions of people worldwide each year (Angus et al., 2001; Dellinger, 2003; Rhodes et al., 2017), and its incidence is not declining (Gaieski et al., 2013). Currently, the pathophysiological basis of sepsis is thought to involve disordered pro- and anti-inflammatory responses, which suggests a new method for the treatment of this deadly condition (Hotchkiss et al., 2013). The prognosis is associated with not only the virulence of the pathogens but also the septic patient’s age and coexisting conditions, such cardiovascular dysfunction (Angus and van der Poll, 2013).

Calcium channel blockers (CCBs) are widely administered for the treatment of cardiovascular disease, including hypertension and ischaemic heart disease (Fihn et al., 2012; James et al., 2014). These drugs inhibit Ca^2+^ channels in the myocardium and vascular smooth muscle cells, resulting in the inhibition of myocardial contractions, the pulse conduction system (anti-arrhythmias), and vasodilation (Sueta et al., 2017). Cardiovascular disease is well known to be one of the most common coexisting conditions in septic patients and is independently related to an increased risk of death during hospitalization (Martin et al., 2003; Vincent et al., 2009). Sepsis is related to an overload of Ca^2+^ in many cell types (Hotchkiss and Karl, 1996) that can lead to disordered cellular processes, cytotoxicity or even cell death *via* a variety of mechanisms, such as metabolic manifestations, vascular smooth muscle tone dysregulation, mitochondrial dysfunction, nuclear damage, cytoskeletal breakage, the production of nitric oxide and pro-inflammatory cytokines, and apoptosis (Dong et al., 2006; Clapham, 2007). However, CCBs can restore such disrupted cellular processes to their normal states through calcium channel-dependent calcium ion homeostasis. Furthermore, CCBs exert pleiotropic effects, such as antioxidant effects (Mason et al., 1998) and immunodepression and anti-inflammatory activity suppression (Das et al., 2009), in sepsis. Therefore, CCB use may benefit patients with sepsis.

Recently, Wiewel et al. (2017) reported that previous CCB use conferred an obvious survival benefit compared with non-CCB use in patients with sepsis. However, several studies (Lee et al., 2017; Kim et al., 2019; Hsieh et al., 2020) have indicated that prior CCB use was not related to lower mortality in septic patients. In addition, Hsieh et al. (2020) reported that CCBs were associated with decreased 30-days mortality in septic shock patients. However, de Roquetaillade et al. (2020) indicated that CCB use was not associated with lower mortality in septic shock patients. Therefore, the relationship between previous CCB use and prognosis in sepsis patients is controversial. Thus, the available data were synthesized to evaluate whether CCBs are helpful for reducing mortality in septic patients.

## Materials and Methods

The study protocol is registered on the PROSPERO website (http://www.crd.york.ac.uk/PROSPERO) with the registration number CRD42019127112, and it can be found online at https://www.crd.york.ac.uk/PROSPERO/display_record.php?RecordID=127112.

### Search Strategy

The meta-analysis of observational studies in epidemiology (MOOSE) guidelines were followed (Stroup et al., 2000). In addition, the PRISMA 2009 checklist is shown in Supplementary Table S1. A comprehensive literature search for cohort studies on the association between CCB use and mortality in septic patients published from the dates of database inception to April 9, 2020, was performed in the MEDLINE (www.ncbi.nlm.nih.gov/pubmed), EMBASE (www.embase.com), Cochrane CENTRAL (https://www.cochranelibrary.com/central), and Web of Science (https://apps.webofknowledge.com) databases. A combination of MeSH/Emtree terms and title, abstract or keyword terms was used. The search terms were “calcium channel blockers,” “calcium channel blocking agent,” “calcium antagonist,” “sepsis,” and “septic shock.” The detailed retrieval strategy can be seen in Supplementary Table S2. The search was restricted to studies published in English. Furthermore, we reviewed the references of eligible articles to identify other potentially relevant studies. The literature searches were conducted independently by Xianfei Ding and Yuqing Cui.

### Eligibility Criteria

Studies were considered eligible for inclusion in the meta-analysis if they met the following population, intervention, comparators, outcomes and study design (PICOS) criteria: 1) the population included adult sepsis and/or septic shock patients, 2) the intervention involved the prior use of CCBs, 3) the comparison was with no prior use of CCBs, 4) the primary outcome was 90-days mortality, or secondary outcome was short-term (30-days, Intensive Care Unit (ICU), in-hospital) mortality, 5) the study design was an observational cohort study. We excluded relevant studies that did not report the mortality of sepsis or septic shock patients. In addition, we also excluded studies for which full texts could not be obtained and summary and review articles.

### PICOS Question

Population: Adult sepsis and/or septic shock patients.
Intervention: Prior use of CCB.
Comparison: No prior use of CCB.
Outcome: Mortality.
Study design: Prospective observational or retrospective cohort studies.

### Study Selection and Data Extraction

Xianfei Ding and Yuqing Cui independently screened the titles and/or abstracts of all retrieved studies to determine whether they met the eligibility criteria and noted the reason for the exclusion of each rejected article (Kappa statistic = 0.91). Key data explications were performed independently by Huoyan Liang and Lifeng Li. All disputes were settled by discussions among Dong Wang, Quancheng Kan, and Lexin Wang. The following characteristics were extracted from all the included studies: first author, year (publication), country, study design, CCB and non-CCB use in patients with sepsis, sex composition of patients, study duration, and unadjusted or adjusted odds ratios (ORs) with 95% confidence intervals (CIs) for the primary and secondary outcomes.

### Assessment of Risk of Bias

The risk of bias of the eligible studies was evaluated with the Newcastle-Ottawa Scale (NOS) for cohort studies (Wells and O’Connell, 2014). A maximum of nine points could be obtained: four points was the maximum for selection, two points was the maximum for design and analysis comparability, and three points was the maximum for the assessment of outcomes. High-quality studies received a score ≥7, whereas moderate- and low-quality studies received scores of 4–6 and ≤4, respectively. The Grading of Recommendations, Assessment, Development and Evaluation (GRADE) criterion was used to estimate and summarize the quality of the evidence (Brozek et al., 2009).

### Statistical Analysis

For binary data, we used ORs and their 95% CIs to estimate the effect sizes of our outcome of interest. The pooled ORs from the included studies were calculated with a random-effects model, and the *I*-*V* heterogeneity method was used to generate the forest plots. Heterogeneity (Higgins et al., 2003) among studies was evaluated by the *I* ^2^ statistic; *I* ^2^ values of 0–25% represented no heterogeneity, values of 25–50% represented slight heterogeneity, values of 50–75% represented moderate heterogeneity, and values of 75–100% represented high heterogeneity. Begg’s funnel plot (Begg and Mazumdar, 1994) was constructed, and Egger’s linear regression (Stuck et al., 1998) was performed to evaluate potential publication bias. Funnel plots (Hunter et al., 2014) were visually evaluated for asymmetry. One-way sensitivity analysis (Copas and Shi, 2001) was performed to evaluate the robustness of the results. All statistical analyses were performed with Stata 14.0 (College Station, TX, 77,845, United States, Serial number: 401406267051).

## Results

### Study Selection

**FIGURE 1:**
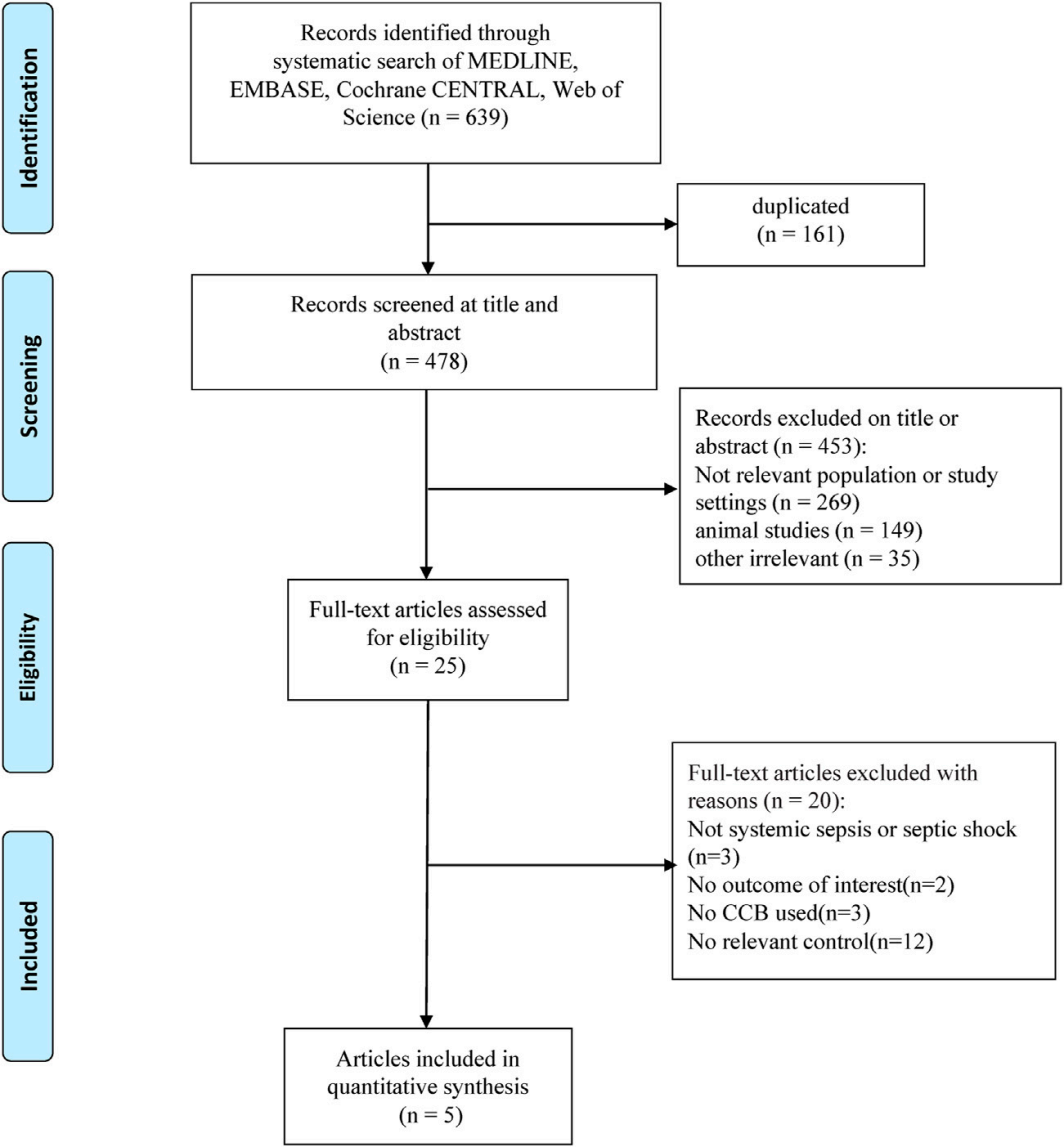
Flow chart of the literature selection process.

The initial literature search yielded 639 potentially relevant publications, and 478 records remained after removing duplicates. We then excluded 453 records after the preliminary title and abstract screening. After evaluating the full texts of the remaining 25 records, we identified five cohort studies (Lee et al., 2017; Wiewel et al., 2017; Kim et al., 2019; de Roquetaillade et al., 2020; Hsieh et al., 2020) that were eligible for inclusion in this meta-analysis (Figure 1).

### Study Characteristics

A detailed description of the five included studies is shown in Table 1. In total, 280,982 septic patients were included in this meta-analysis. All the included studies were multi-centre cohort studies that involved septic patients who reported their prior use of CCBs (Lee et al., 2017; Wiewel et al., 2017; Kim et al., 2019; de Roquetaillade et al., 2020; Hsieh et al., 2020). We extracted the adjusted or propensity-matched ORs and 95% CIs from the primary and secondary outcome data. Otherwise, the data were calculated from the raw data in each included study.

**TABLE 1:** Summary of identified studies.

Author/Year	Country	Study Design	Study Duration		Female/Male	Descriptions of participants	Disease severity	Number of Patients in CCB(death)/non-CCB Use(death)	Follow up	90-day Mortality in sepsis^a^ (OR, 95% CI)	Short-term Mortality in septic shock (OR, 95% CI)	Short-term Mortality in sepsis^a^ (OR, 95% CI)	Comorbidities
Wiewel et al. (2017) (18)	Netherlands	PC	2011/7–2013/7	420/640		Critical sepsis	SOFA 7 (5–9)/7 (5–9)	197(58)/863(326)	90-day	0.62 (0.40–0.96)	0.31 (0.14–0.65)	0.48 (0.31–0.74)^a^	Cerebrovascular disease Chronic cardiovascular insufficiency Chronic renal insufficiency Congestive heart failure Chronic obstructive pulmonary disease Diabetes mellitus Hematologic malignancy Hypertension Immune deficiency Metastatic malignancy Myocardial infarction Nonmetastatic malignancy Peripheral vascular disease
Lee et al. (2017) (19)	Taiwan	RC	2000–2011	20,903/30,175		Sepsis	Average number of organ dysfunction 1 (1–2)/1 (1–2)	19,742/31,336	90-day	0.91 (0.85–0.97)	NA	0.92 (0.85–0.99)	Myocardial infarction Congestive heart failure Peripheral vascular disease Cerebrovascular disease Dementia Chronic pulmonary disease Rheumatologic disease Peptic ulcer disease Diabetes with chronic complications Hemiplegia or paraplegia Renal disease
Kim et al. (2019) (20)	South Korea	RC	2003–2013	2,328/2,221		Sepsis	NA	1,287(586)/3,262(1,583)	90-day	0.89 (0.78–1.01)	NA	0.83 (0.72–0.95)	Cardiovascular Chronic respiratory disease Chronic renal disease Chronic liver disease Diabetes Cerebrovascular Solid tumor Hematologic disease
Hsieh et al. (2020) A (21)	Taiwan	RC	1999–2013	NA		Sepsis	NA	NA	28-day	NA	NA	1.21 (1.17–1.26)^b^	Hyperlipidemia Congestive heart failure chronic kidney disease chronic liver disease chronic obstructive pulmonary disease ischemic heart disease Hypertension Cancer
Hsieh et al (2020) B (21)	Taiwan	RC	1999–2013	NA		Septic shock	NA	NA	28-day	NA	0.64 (0.53–0.77)	NA	
Roquetaillade et al. (2020) (22)	French	RC	2008–2016	NA		Septic shock	SOFA 9.57 (3.71)/10.96 (4.06)	103/632	ICU	NA	0.95 (0.52–1.74)	NA	Chronic heart failures Arterial hypertension Diabetese Obesity Cirrhosis Chronic obstructive pulmonary disease chronic kidney failure Imunosuppression

Abbreviations: PC, prospective cohort; RC, retrospective cohort; SOFA, Sequential Organ Failure Assessment; CCB, calcium channel blockers; NA, not available; OR, odds ratio; CI, confidence interval.

^a^30-day Mortality in sepsis that included septic shock.

^b^30-day Mortality in sepsis that not included septic shock.

### Risk of Bias Assessment

The risk of bias assessment of the included studies is shown in Table 2. The five eligible observational cohort studies (Lee et al., 2017; Wiewel et al., 2017; Kim et al., 2019; de Roquetaillade et al., 2020; Hsieh et al., 2020) had scores ≥8 and were considered to have a low risk of bias according to the NOS.

**TABLE 2:** The Newcastle-Ottawa Scale (NOS) for assessing the quality of including studies.

Studies First Author		Selection					Comparability			Assessment of outcome				Total Quality Score
		Representativeness of CCB Use Arm(s)	Selection of the non-CCB Use Arm(s)	Origin of Exposure Source	Demonstration that Outcome of Interest was not Present at Start of Study		Studies Controlling the Most Important Factors	Studies Controlling the Other Main Factors		Assessment of Outcome with Independency	Adequacy of Follow-up Length (to assess outcome)	Lost to Follow-up Acceptable (less than 10% and reported)		
Wiewel et al. (2017) (18)		*	*	*	*		*	*		*	*	*		9
Lee et al. (2017) (19)		*	*	*	*		*	*		*	*			8
Kim et al. (2019) (20)		*	*	*	*		*	*		*	*			8
Hsieh et al. (2020) (21)		*	*	*	*		*	*		*	*			8
Roquetaillade et al. (2020) (22)		*	*	*	*		*	*		*	*			8

Abbreviations: CCB calcium channel blockers. Each star represents reaching the standard, and starred items are given one point.

### Effects of CCB on Septic Patients

**FIGURE 2:**
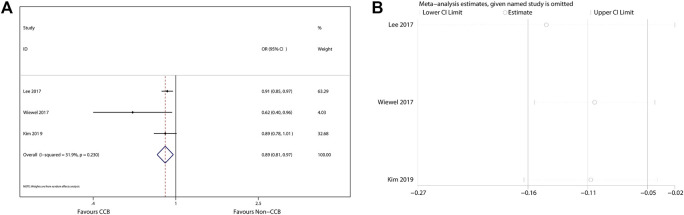
**(A)** Forest plot showing the significance of the relationship between the prior use of CCBs and 90-days mortality in patients with sepsis according to the random-effects model. **(B)** The sensitivity analysis showed that the studies were robust and reliable with regard to the relationship between prior CCB use and 90-days mortality in patients with sepsis.

**FIGURE 3:**
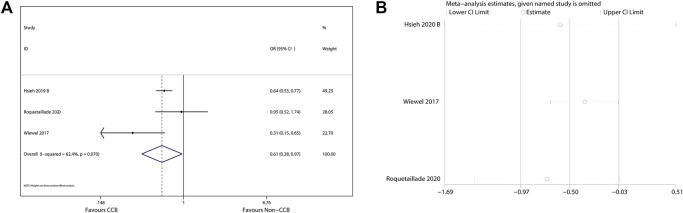
**(A)** Forest plot showing the significance of the relationship between the prior use of CCBs and short-term mortality in patients with septic shock according to the random-effects model. **(B)** The sensitivity analysis showed that the studies were robust and reliable for the association of prior CCB use with short-term mortality in patients with septic shock.

**FIGURE 4:**
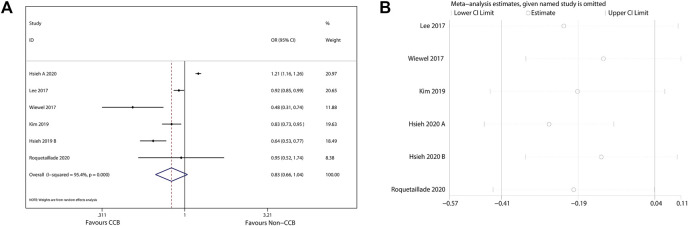
**(A)** Forest plot showing the significance of the relationship between the prior use of CCBs and short-term mortality in patients with sepsis according to the random-effects model. **(B)** The sensitivity analysis showed that the studies were robust and reliable with regard to the relationship between prior CCB use and short-term mortality in patients with sepsis.

The 90-days mortality rate, which was the primary outcome, and the short-term mortality rates, which were the secondary outcomes, are shown in Figures 2A–4A. Prior CCB use was associated with a significantly reduced 90-days mortality rate in sepsis patients [OR, 0.90 (0.85–0.95); *I* ^2^ = 31.9%; evidence rank, very low] (Figure 2A). Moreover, prior CCB use was associated with a reduced short-term mortality rate in septic shock patients [OR, 0.61 (0.38–0.97); *I* ^2^ = 62.4%; evidence rank, very low] but not in sepsis patients [OR, 0.83 (0.66–1.04); *I* ^2^ = 95.4%; evidence rank, very low] (Figures 3A, 4A).

### Sensitivity Analysis

As the included studies were observational cohort studies with a low risk of bias (Table 2), a sensitivity analysis of the methodological criteria was not conducted. A sensitivity analysis was conducted to evaluate the effect of any one study on the pooled ORs and 95% CIs by removing one individual study at a time. The sensitivity analysis findings indicated that the results were robust and reliable (Figures 2B–4B).

### Publication Bias

Because the number of included studies that reported the effects of CCB use on septic patients was small (<10), we did not generate a funnel plot, as it may not have discovered publication bias (Lau et al., 2006).

## Discussion

This meta-analysis involving 280,982 patients indicated that compared with no prior CCB use, prior CCB use was related to a reduced 90-days mortality rate in patients with sepsis and a reduced short-term mortality rate in patients with septic shock. To our knowledge, this is the first meta-analysis to explore and evaluate the relationship between prior CCB use and mortality in septic patients. These findings indicate that CCB administration is associated with significant improvements in the 90-days prognosis of sepsis patients and the short-term survival of septic shock patients.

Currently, the effect of prior CCB use on the mortality of septic patients remains unclear (Lee et al., 2017; Wiewel et al., 2017; Kim et al., 2019; de Roquetaillade et al., 2020; Hsieh et al., 2020). Several animal studies (Németh et al., 1998; Wyska, 2009) have suggested that CCBs could reduce mortality in endotoxaemic mouse models. Verapamil improved the survival rate of dogs with endotoxic shock (Bosson et al., 1985), and nifedipine increased survival in a bacteraemia model (Bosson et al., 1986). However, the results of clinical studies are not consistent with the results of these animal studies (Lee et al., 2017; Kim et al., 2019; Hsieh et al., 2020). This meta-analysis provides evidence supporting the association of prior CCB use with decreased mortality in patients with sepsis.

The potential mechanism underlying the association of CCB use and mortality in septic patients remains unclear. CCB may ameliorate cardiac dysfunction (Bosson et al., 1985; Zhu et al., 2005) in septic survivors with cardiovascular complications (Ou et al., 2016). A previous meta-analysis showed that prior β-blocker use was associated with a reduction in mortality, which may be due to decreased cardiac systolic and diastolic dysfunction (Tan et al., 2019). Several studies have reported that CCBs differentially inhibit the generation of pro-inflammatory factors, such as interleukin-12 (IL-12), interferon-gamma (IFN-γ) (Németh et al., 1998), and TNF-alpha (Li et al., 2009), in sepsis patients. Additionally, CCBs inhibit the nuclear transcription factor NF-κB and activate PI3K/Akt passage (Mustafa and Olson, 1999; Hayashi et al., 2000; Li et al., 2006; Hassoun et al., 2008), which reduce LPS-induced acute inflammatory reactions (Zhang et al., 2007). Moreover, CCBs have been shown to lower oxidative burst and inducible nitric oxide synthase (iNOS) protein expression to regulate the inflammatory response (Hotchkiss et al., 1997) and ameliorate cellular injury and cardiac dysfunction. Most importantly, sepsis disrupts intracellular calcium homeostasis, which leads to endothelial injury and destroys subcellular structures (Duchen, 2000; Ding et al., 2013). CCBs, which are involved in targeting and blocking calcium ion overload (Meldrum et al., 1993; Song et al., 1993), could reduce intracellular Ca^2+^ levels and prevent cytotoxicity. However, the relationship between CCB administration and an improved prognosis of sepsis needs to be confirmed in clinical trials.

A meta-analysis is used to systematically and statistically analyze a variety of studies on the same topic. The summarized meta-analysis results have significant heterogeneity when the differences among outcomes in the included individual studies are greater than expected. In the present meta-analysis, the assessment of the risk of bias in the included studies showed a low risk of bias; thus, methodological heterogeneity did not exist.

This meta-analysis has a number of advantages. First, the sample of included septic patients was large, suggesting that the results may be stable. The large population was sufficient to conduct propensity score matching, which is a method of reducing the effects of deviations and confounding variables between the CCB and the non-CCB groups. Second, the NOS was used to assess the risk of bias. The results indicated that the studies that met the inclusion criteria for this meta-analysis had a low risk of bias. Third, we extracted the adjusted or propensity-score matched ORs and 95% CIs to calculate the pooled ORs for the effect of CCB use on mortality in an unbiased manner. Fourth, the sensitivity analysis suggested that the results were robust and reliable.

However, this meta-analysis has several limitations. Although we conducted an overall search to identify the pertinent studies as far back as possible, only five studies were included; more research may be needed to confirm this conclusion. Nevertheless, the robustness of the conclusion was supported by the sensitivity analysis. In addition, our study was limited by high heterogeneity. According to the inclusion criteria for each study, there were differences in racial and other characteristics of the participants and the timing and use of antihypertensive drugs, leading to high heterogeneity. In addition, the studies included in this meta-analysis were only observational studies, not randomized controlled trials. A certain limitation exists even if all the included cohort studies show a low risk of bias. The effectiveness of prior CCB treatment on mortality in sepsis and septic shock patients needs to be further investigated in high-quality studies.

## Conclusion

This is the first systematic review and meta-analysis to report the association between prior CCB use and mortality in septic patients. We discussed the effects of prior CCB use on cardiovascular function and inflammation in sepsis. This meta-analysis suggests that prior CCB use is significantly associated with improvements in the 90-days prognosis of sepsis patients and the short-term survival of septic shock patients. However, this finding should be evaluated in future randomized controlled trials. CCBs remain an attractive potential method for the improvement of sepsis-related mortality.

## Data Availability

The original contributions presented in the study are included in the article/Supplementary Material, further inquiries can be directed to the corresponding author.

## Abbreviations

CCB, calcium channel blocker; CIs, confidence intervals; NOS, Newcastle-Ottawa Scale; PICOS, population, intervention, comparators, outcomes and study design; LPS, lipopolysaccharide.
