# Supplementary material for: Association Between Prior Calcium Channel Blocker Use and Mortality in Septic Patients: A Meta-Analysis of Cohort Studies
Source: Front Pharmacol. 2021 May 25;12:628825. doi: 10.3389/fphar.2021.628825 (PMC8185201; doi:10.3389/fphar.2021.628825)
Supplement: Supplementary file 2 [file Table2.docx]

**Supplemental Table 2. Detailed search strategy.**

| **Electronic databases** | **Search** | **Search strategy** | **Results** |
| --- | --- | --- | --- |
| **MEDLINE** | #1 | ("Calcium Channel Blockers"[MeSH Terms]) OR "Calcium Channel Blockers"[Title/Abstract] | 40739 |
|  | #2 | ("Bepridil"[MeSH Terms]) OR "Bepridil"[Title/Abstract] | 987 |
|  | #3 | #1 OR #2 | 41400 |
|  | #4 | ("Nitrendipine"[MeSH Terms]) OR "Nitrendipine"[Title/Abstract] | 3135 |
|  | #5 | #3 OR #4 | 43106 |
|  | #6 | ("Shock, Septic"[MeSH Terms]) OR "Sepsis"[MeSH Terms] | 115033 |
|  | #7 | ("Sepsis"[Title/Abstract]) OR "septic shock"[Title/Abstract] | 100700 |
|  | #8 | #6 OR #7 | 173423 |
|  | #9 | #5 AND #8 | **77** |
| **EMBASE** | #1 | 'sepsis'/de OR 'septic shock'/de OR 'pyaemia' | 180222 |
|  | #2 | 'sepsis':ti,ab,kw OR 'septic shock':ti,ab,kw OR 'pyaemia':ti,ab,kw | 155402 |
|  | #3 | #1 OR #2 | 225277 |
|  | #4 | 'calcium channel blocking agent'/de OR 'calcium channel blockers'/de OR 'calcium antagonist'/de OR 'bepridil'/de OR 'nitrendipine'/de | 83803 |
|  | #5 | 'calcium channel blocking agent':ti,ab,kw OR 'calcium channel blockers':ti,ab,kw OR 'calcium antagonist':ti,ab,kw OR 'bepridil':ti,ab,kw OR 'nitrendipine':ti,ab,kw | 23707 |
|  | #6 | #4 OR #5 | 91245 |
|  | #7 | #3 AND #6 | **467** |
| **Cochrane CENTRAL** | #1 | MeSH descriptor: [Sepsis] explode all trees | 3970 |
|  | #2 | MeSH descriptor: [Shock, Septic] explode all trees | 763 |
|  | #3 | (sepsis):ti,ab,kw | 8082 |
|  | #4 | (septic shock):ti,ab,kw | 2238 |
|  | #5 | #1 or #2 or #3 or #4 | 10670 |
|  | #6 | MeSH descriptor: [Calcium Channel Blockers] explode all trees | 2790 |
|  | #7 | MeSH descriptor: [Bepridil] explode all trees | 44 |
|  | #8 | MeSH descriptor: [Nitrendipine] explode all trees | 350 |
|  | #9 | (calcium channel blocking agent):ti,ab,kw | 1362 |
|  | #10 | (calcium channel blockers):ti,ab,kw | 4154 |
|  | #11 | (calcium antagonist):ti,ab,kw | 3758 |
|  | #12 | (bepridil):ti,ab,kw | 91 |
|  | #13 | (nitrendipine):ti,ab,kw | 558 |
|  | #14 | #6 or #7 or #8 or #9 or #10 or #11 or #12 or #13 | 7126 |
|  | #15 | #5 and #14 | **8** |
| **Web of Science** | #1 | TOPIC: (" Calcium Channel Blockers ") | 47128 |
|  | #2 | TOPIC: ("sepsis") | 194591 |
|  | #3 | #1 AND #2 | **85** |
